# Prevalence and antimicrobial susceptibilities of bacterial pathogens in Chinese pig farms from 2013 to 2017

**DOI:** 10.1038/s41598-019-45482-8

**Published:** 2019-07-09

**Authors:** Bingzhou Zhang, Xugang Ku, Xuexiang Yu, Qi Sun, Hao Wu, Fangzhou Chen, Xiaoqian Zhang, Long Guo, Xibiao Tang, Qigai He

**Affiliations:** 1 Division of Animal Infectious Diseases, State Key Laboratory of Agricultural Microbiology, College of Animal Sciences and Veterinary Medicine, Huazhong Agricultural University, Wuhan, 430070 P.R. China; 2 The Diagnostic Center for Animal Disease of Huazhong Agricultural University, Wuhan, 430070 P.R. China

**Keywords:** Cellular microbiology, Bacterial pathogenesis

## Abstract

Bacterial diseases of swine are a kind of multifactorial and uncontrollable diseases that commonly exist in pig farms all over the world and will lead to huge economic losses every year. In this study, a detailed and overall survey was carried out to better understand the prevalence and antimicrobial susceptibilities of bacterial diseases from 2013 to 2017 in China. A total of 19673 bacterial strains were isolated from 44175 samples collected from 9661 pig farms that distributed in 16 Chinese major pig breeding provinces. The results showed that the average isolation rates of *Streptococcus suis* (SS), *Haemophilus parasuis* (HPS), *Escherichia coli* (*E. coli*), *Pasteurella multocida* (Pm), *Actinobacillus pleuropneumoniae* (APP), *Brodetella bronchiseptica* (Bb), *Salmonella enteria* (SE), *Erysipelothrix rhusiopathiae* (E. rhusiopathiae) were 16.9%, 9.7%, 6.3%, 3.4%, 0.3%, 1.5%, 2.3% and 0.9%, respectively. The isolate rates of *E. coli*, APP and SE showed an increasing trend from 2013 to 2017. The seasonal prevalence characteristics of SS, HPS and Pm were obviously higher from April to August for first two bacteria and higher at February, March, April, and October for Pm. The dominant serotypes for SS, HPS were serotype 2 and serotype 5 (changed from serotype 4), respectively. The SS, HPS, and Pm showed very high antibiotic resistance rates to almost 8 common antibiotics (β-lactam, aminoglycoside, macrolides, lincomycin, tetracycline, quinolone, polymyxin, and sulfonamide) and an obvious increasing trend of antibiotic resistance rates from 2013 to 2017. In conclusion, the study provides detailed information on the prevalence and antimicrobial susceptibilities of different bacterial pathogens of swine from 2013 to 2017 in China. These data can provide a foundation for monitoring epidemiological patterns of bacterial diseases in the Chinese swine herds, as well as provide insight into potential antibiotic resistance profiles in these pathogens.

## Introduction

Bacterial diseases heavily affect the health of swine, especially, respiratory system and digestive system diseases which are reported to be associated with intensive pig production^1^. For example, *Streptococcus suis* (SS) can lead to septicemia, meningitis, arthritis, acute death and even affect the health of human^2^. *Haemophilus parasuis* (HPS) and *Actinobacillus pleuropneumoniae* (APP) can lead to dyspnea, pneumonia, pleurisy, and progressive emaciation^3^. *Escherichia coli* (*E. coli*) and *Salmonella enteria* (SE) can lead to diarrhea and other gastrointestinal diseases^4,5^. *Pasteurella multocida* (Pm) and *Brodetella bronchiseptica* (Bb) can together lead to swine atrophic rhinitis^6^. Meanwhile, *Erysipelothrix rhusiopathiae* (E. rhusiopathiae) can lead to cutaneous necrosis, endocarditis, and arthritis^7^. In total, they are all the reasons that heavily affect the health of swine and lead to economic losses of pig industry.

Because of their complexity and indeterminacy, bacterial diseases are very difficult to control. Among them, serotype is a very important factor, we know that bacteria often have many kinds of serotypes and they often lack cross-protection between different serotypes. Furthermore, serotype has obvious distribution difference between different regions. For example, the main serotypes of SS in Canada are serotype 2, 1/2, 3 and 4^8^. However, serotype 2, 9, 7 SS are dominant in China. Therefore, it is very important to know the main serotypes of common bacterial pathogens for controlling bacterial diseases and it will also supply guidance for developing vaccines.

Antimicrobial agents are widely used in bacterial diseases of swine, especially, respiratory tract infections and diarrheal diseases. Antibiotics have been widely used in livestock industries since the early 1990s in China. The average usage of veterinary antibiotics has reached approximately 6000 tons annually^9^ and most of them are used as feed additives, such as tetracyclines, sulfonamides, fluoroquinolones, macrolides and others^10^. The widely use of veterinary antibiotics greatly contributes to the development of livestock industries. However, there are also some problems existed in usage of antibiotics, such as abusing of antibiotics in fodder, drinking water, and injection and violating withdrawal time, which all will lead to an increase in antibiotic resistance rates^10,11^. In addition, it not only affects the development of the pig industry but also threats the health of human^12^. Therefore, how to improve the production efficiently and decrease the use of antibiotics have become urgent and difficult problems.

Though significant progress has been made in the last few decades in reducing the prevalence of bacterial diseases, there is still an increasing concern over the losses associated with diseases. So, the isolation rates, regional and seasonal distribution, serotype survey, and antimicrobial susceptibilities of major bacteria were analyzed to understand, prevent and control bacterial diseases in China.

## Materials and Methods

### Samples collection

**Figure 1:**
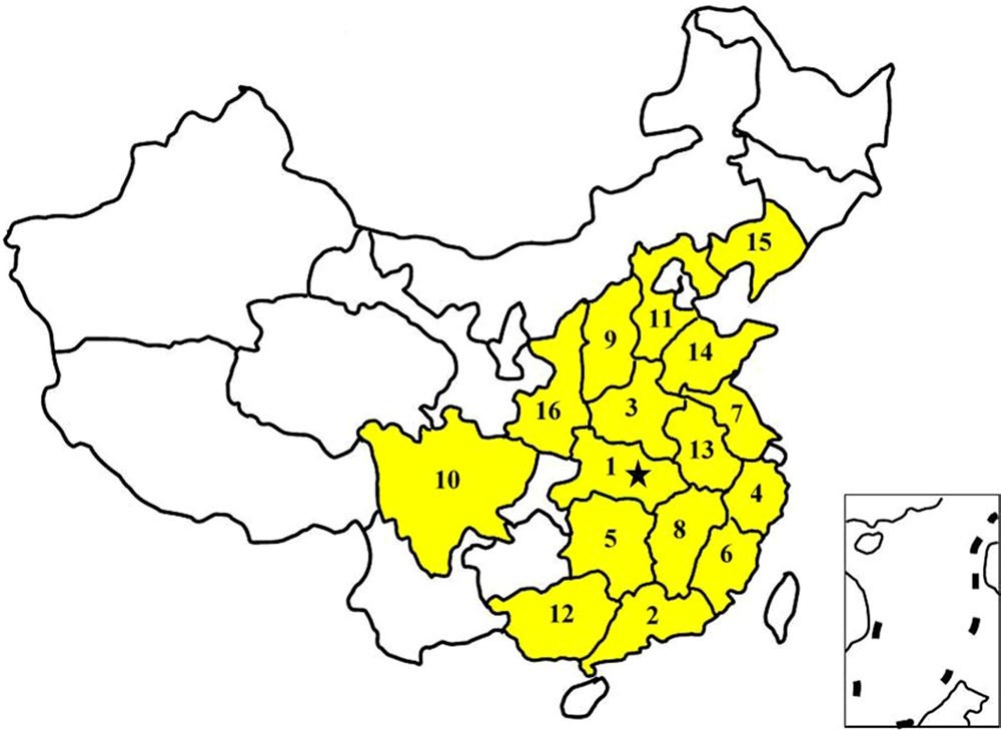
Geographic distribution of different collected samples. The position of asterisk is the location of our diagnostic laboratory. 1–16 represented different provinces where samples were collected and the ranking of the number of samples. 1: Hubei (12897, 29.2%), 2: Guangdong (12389, 28.0%), 3: Henan (5890, 13.3%), 4: Zhejiang (3902, 8.8%), 5: Hunan (3080, 7.0%), 6: Fujian (960, 2.2%), 7: Jiangsu (897, 2.0%), 8: Jiangxi (853, 1.9%), 9: Shanxi (696, 1.6%), 10: Sichuan (652, 1.5%), 11: Hebei (533, 1.2%), 12: Guangxi (295, 0.7%), 13: Anhui (263, 0.6%), 14: Shandong (257, 0.6%), 15: Liaoning (157, 0.4%), 16: Shanxi (119, 0.3%) and others (332, 0.8%).

**Figure 2:**
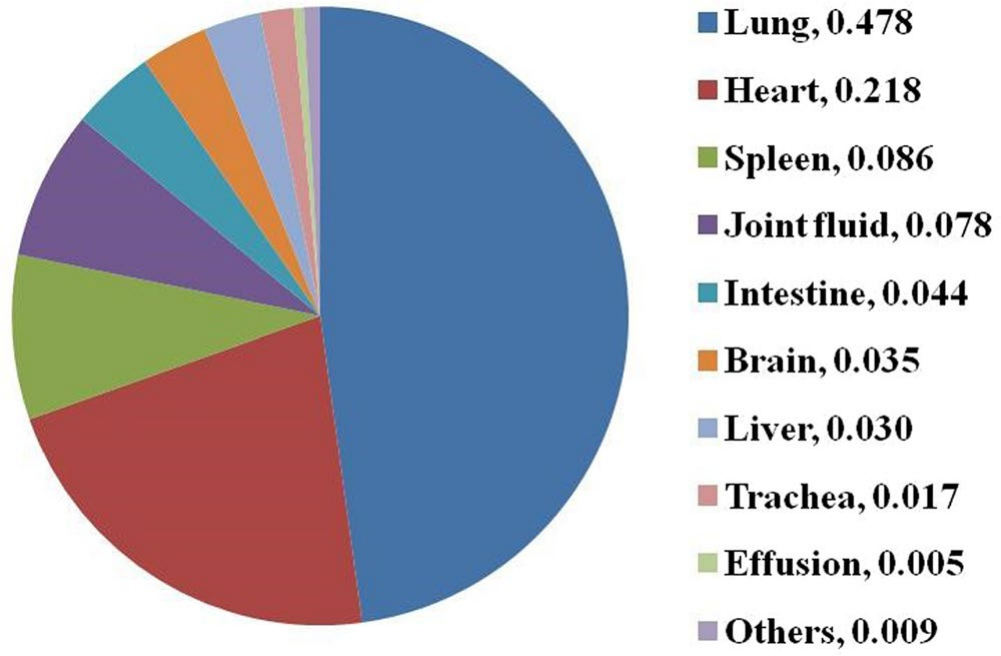
Tissue sources of collected samples. Tissue sources of these samples were lung (21127, 47.8%), heart (9612, 21.8%), spleen (3801, 8.6%), joint fluid (3445, 7.8%), intestine (1942, 4.4%), brain (1556, 3.5%), liver (1314, 3.0%), trachea (765, 1.7%), effusion (235, 0.5%) and others (378, 0.9%).

All pig tissue samples were collected from 16 Chinese major pig breeding provinces from 2013 to 2017 and delivered to the Animal Disease Diagnostic Center of Huazhong Agricultural University for identification of bacterial pathogens, which is a reference lab in China where people send pig samples for diagnosis (Fig. 1). All samples that were sent to our reference lab with a total of 44175 samples from 9661 pig farms were collected and chosen for bacterial isolation and identification (Table 1). The pig farms had a wide variety of management types and herd size with small-, medium- and large-scale commercial pig farms as well as various types of backyard farms. The tissues came from suspected sick pigs and were transported to our reference lab. The collected samples included lung, heart, spleen, joint fluid, intestine, brain, liver, trachea, effusion and so on (Fig. 2). Then, sterile operation had been taken to avoid cross contamination and all samples were processed for bacterial isolation immediately. The research was approved by the Ethics Committee of the Faculty of Veterinary Medicine of the Huazhong Agricultural University. All procedures regarding the animal care and testing were carried out according to the recommendation of Hubei provincial public service facilities.

**Table 1:** Samples information.

Years	No. pig farms	No. samples	No. isolated bacteria
2013	1374	7496	3116
2014	2989	9452	3926
2015	2974	9320	4495
2016	1051	8226	3543
2017	1273	9681	4593
Total	9661	44175	19673

### Culture conditions, primers, and identification methods

Common bacterial pathogens were isolated and identified from Chinese pig farms, such as SS, HPS, Pm, *E. coli*, APP, Bb, SE, E. rhusiopathiae, and others. Tryptic Soy Broth (TSB), Tryptic Soy Agar (TSA) (Difco Laboratories, Detroit, USA), Shigella and Salmonella Agar and MacConkey Agar (HuanKai Microbial, Guangdong, China) medium were used and 10 μg/mL of nicotinamide adenine dinucleotide (NAD) and 5% (v/v) inactivated cattle serum (Zhejiang Tianhang Biotechnology, Zhejiang, China) were added if necessary. Primers used for identification and serotyping were listed in Table 2 and Supplementary Table 1, respectively.

**Table 2:** Primers used in this study.

Strains	Gene	Name	Sequence (5′ → 3′)	Amplicons size (bp)	References
*Streptococcus suis*	*gdh*	JP4 JP5	GCA GCGTATTCTGTCAAACG CCATGGACA GATAAA GATGG	689	^47^
*Haemophilus parasuis*	16S rRNA	HPS-1 HPS-2	GGCTTCGTCACCCTCTGT GTGATGAGGAAGGGTGGTGT	822	^48^
*Pasteurella multocida*	kmt1	KMT1T7 KMT1SP6	ATCCGCTATTTACCCAGTGG GCTGTAAACGAACTCGCCAC	457	^49^
*Escherichia coli*	uidA	Ec-1 Ec-2	AAAACGGCAAGAAAAAGCAG GCGTGGTTACAGTCTTGCG	147	^50^
*Actinobacillus pleuropneumoniae*	apxIVA	APXIVA-1 APXIVA-2	TGGCACTGACGGTGATGA GGCCATCGACTCAACCAT	442	^51^
*Brodetella bronchiseptica*	fla	Fla4 Fla2	TGGCGCCTGCCCTATC AGGCTCCCAAGAGAGAAA	237	^52^
*Salmonella enteria*	invA	INVA-1 INVA-2	ACAGTGCTCGTTTACGACCTGAAT AGACGACTGGTACTGATCGATAAT	580	^53^
*Erysipelothrix rhusiopathiae*	ER	ER1 ER2	CGATTATATTCTTAGCACGCAACG TGCTTGTGTTGTGATTTCTTGACG	937	^54^
Universal primer	16S rRNA	27F 1492R	AGAGTTTGATCCTGGCTCAG TACGGCTACCTTGTTACGACTT	1466	^21^

All plates were incubated at 37 °C for 24 to 48 h. After this isolation stage, the strains were identified by colony morphology, Gram-staining characteristics and oxidase (Gram-negative bacilli) or catalase tests. Phenotypic methods or standard biochemical procedures were used to identify suspected bacteria of SS, HPS, Pm, *E. coli*, APP, Bb, SE, E. rhusiopathiae, and others based on the previous studies^4,13–19^. To further confirm the phenotypic and biochemical results, PCR methods of specific genes were used based on the references mentioned in Table 2. When PCR identification results were not consistent with the results of phenotypic or standard biochemical procedures, the strains were further identified by 16S rRNA sequencing^20,21^ (Table 2). All isolated bacteria were freeze-dried and kept at −80 °C.

### Serotype identification of SS and HPS

Parts of isolated SS (273/1149, 231/1469, 168/1719, 136/1357 and 128/1796 strains from 2013 to 2017, respectively) and HPS (331/868, 225/883, 206/1231, 179/570 and 154/711 strains from 2013 to 2017, respectively) strains were randomly chosen to do serotype identification based on previously described methods^22,23^. According to the reports, SS can be divided into 33 serotypes based on the difference of capsular polysaccharide, and HPS can be divided into 15 serotypes based on the difference of capsular loci^22,23^.

### Antimicrobial susceptibility test

Antimicrobial susceptibility test was performed based on the standard Clinical and Laboratory Standards Institute (CLSI) guidelines for susceptibility testing. Disk diffusion (DD) was performed according to CLSI M2 A12 Ed. 12 (2015) standards. Tests were performed according to the manufacturer instructions. Briefly, a sterile cotton-tipped swab was dipped into the bacterial suspension (0.5 McFarland) and streaked in three directions across TSA agar plates containing 10 μg/mL of NAD and 5% (v/v) inactivated cattle serum. The plates were dried for 2–3 min, then disks were placed on the agar surface, which contained common antibiotics that used in Chinese pig farms, such as cephradine (CE, 30 μg), ceftriaxone (CRO, 30 μg), amoxicillin (AML, 10 μg), ampicillin (AMP, 10 μg), streptomycin (S, 10 μg), gentamicin (CN, 10 μg), spectinomycin (SH, 100 μg), kanamycin (K, 30 μg), amikacin (AK, 30 μg), neomycin (N, 30 μg), spiramycin (SP, 100 μg), azithromycin (AZM, 15 μg), lincomycin (MY, 2 μg), clindamycin (DA, 2 μg), doxycycline (DO, 30 μg), ofloxacin (OFX, 5 μg), ciprofloxacin (CIP, 5 μg), enrofloxacin (ENR, 5 μg), polymyxin B (PB, 300 μg) and trimethoprim (W, 1.25 μg) (Oxoid, UK). The plates were incubated for 2 days in an aerobic atmosphere at 37 °C. At the end of the incubation period, the diameters of the zones of growth inhibition were measured and the final reference standard based on CLSI^24,25^.

### Statistical analysis

Statistical analyses were undertaken with SAS version 9.0 (SAS Institute Inc.). Univariate association between variables and isolation rates of different bacteria were determined by using univariate ordinary logistic regression analysis and Chi square test. P < 0.05 was considered to be significant.

## Results

### Sample sources of bacterial pathogens

44175 tissues samples from 9661 pig farms were collected and 19673 bacterial pathogens were isolated from 2013 to 2017 (Table 1). The samples were collected from Hubei (12897, 29.2%), Guangdong (12389, 28.0%), Henan (5890, 13.3%), Zhejiang (3902, 8.8%), Hunan (3080, 7.0%), Fujian (960, 2.2%), Jiangsu (897, 2.0%), Jiangxi (853, 1.9%), Shanxi (696, 1.6%), Sichuan (652, 1.5%), Hebei (533, 1.2%), Guangxi (295, 0.7%), Anhui (263, 0.6%), Shandong (257, 0.6%), Liaoning (157, 0.4%), Shanxi (119, 0.3%) and others (332, 0.8%) (Fig. 1), respectively. Among them, Hubei and Guangdong provinces are the major places of sample sources, which contained over half of them (57.2%, 25287/44175). On the one hand, the tissue sources of these samples were lung (21127, 47.8%), heart (9612, 21.8%), spleen (3801, 8.6%), joint fluid (3445, 7.8%), intestine (1942, 4.4%), brain (1556, 3.5%), liver (1314, 3.0%), trachea (765, 1.7%), effusion (235, 0.5%) and others (378, 0.9%) (Fig. 2), respectively.

### The proportion of isolated bacterial pathogens

**Figure 3:**
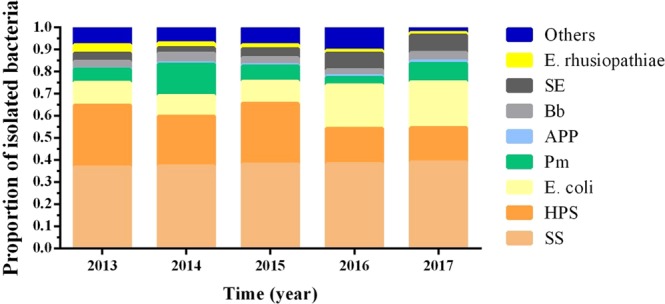
The proportion of isolated bacterial pathogens from 2013 to 2017. Different bacteria were labeled using different colors and the height of the column represented the isolation rates. The successfully isolated bacteria were SS (1149, 36.9%), HPS (868 27.9%), *E. coli* (320, 10.3%), Pm (188, 6.0%), APP (9, 0.3%), Bb (101, 3.2%), SE (117, 3.8%), E. rhusiopathiae (117, 3.8%), and others (247, 7.9%) in 2013, SS (1469, 37.4%), HPS (883, 22.5%), *E. coli* (367, 9.3%), Pm (556, 14.2%), APP (23, 0.6%), Bb (173, 4.4%), SE (94, 2.4%), E. rhusiopathiae (85, 2.2%), and others (276, 7.0%) in 2014, SS (1719, 38.2%), HPS (1231, 27.4%), *E. coli* (451, 10.0%), Pm (309, 6.9%), APP (27, 0.6%), Bb (143, 3.2%), SE (185, 4.1%), E. rhusiopathiae (82, 1.8%), and others (348, 7.7%) in 2015, SS (1357, 38.3%), HPS (570, 16.1%), *E. coli* (694, 19.6%), Pm (120, 3.4%), APP (28, 0.8%), Bb (91, 2.6%), SE (269, 7.6%), E. rhusiopathiae (46, 1.3%), and others (368, 10.4%) in 2016, SS (1796, 39.1%), HPS (711, 15.5%), *E. coli* (955, 20.8%), Pm (379, 8.3%), APP (51, 1.1%), Bb (176, 3.8%), SE (362, 7.9%), E. rhusiopathiae (55, 1.2%), and others (108, 2.4%) in 2017.

The isolation rates of different bacterial pathogens were presented at Fig. 3. From the results, we knew that 8 kinds of common bacteria (SS, HPS, *E. coli*, Pm, APP, Bb, SE, and E. rhusiopathiae) were isolated from pigs and the top three kinds of bacteria were SS, HPS and *E. coli*, which contained about 73.9% of isolated bacteria.

### The prevalence characteristics of different bacterial pathogens

**Figure 4:**
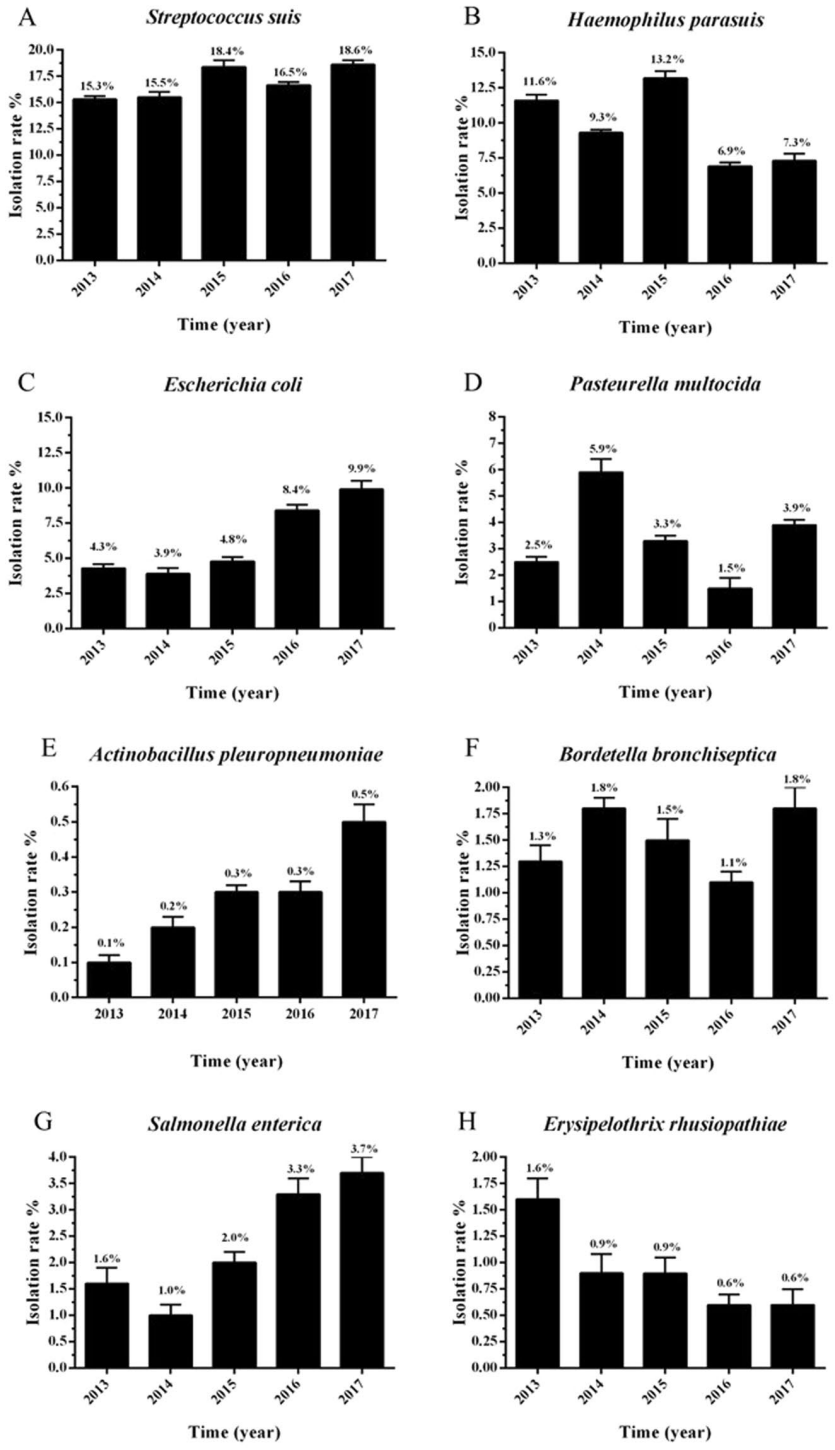
The isolation rates of different bacterial pathogens from 2013 to 2017. (**A**–**H**) represented the isolation rates of SS (**A**), HPS (**B**), *E. coli* (**C**), Pm (**D**), APP (**E**), Bb (**F**), SE (**G**), E. rhusiopathiae (**H**), respectively. Error bars represented the standard deviation of isolation rates of 12 months.

Based on the isolation rates of different bacterial pathogens, the prevalence characteristic of single-pathogen was analyzed (Fig. 4). The detail prevalence trends of SS (Fig. 4A), HPS (Fig. 4B), *E. coli* (Fig. 4C), Pm (Fig. 4D), APP (Fig. 4E), Bb (Fig. 4F), SE (Fig. 4G), E. rhusiopathiae (Fig. 4H) were shown in Fig. 4. From the results, we knew that the prevalence of SS (from 15.3% to 18.6%), *E. coli* (from 3.9% to 9.9%), APP (from 0.1% to 0.5%) and SE (from 1.0% to 3.7%) increased from 2013 to 2017. However, the prevalence of HPS (from 13.2% to 6.9%) and E. rhusiopathiae (from 1.6% to 0.6%) declined from 2013 to 2017. Different with other bacterial pathogens, Pm and Bb showed no significant prevalence trends from 2013 to 2017. But Pm and Bb exhibited a similar changing trend among different years.

### The seasonal prevalence characteristics of SS, HPS, and Pm

**Figure 5:**
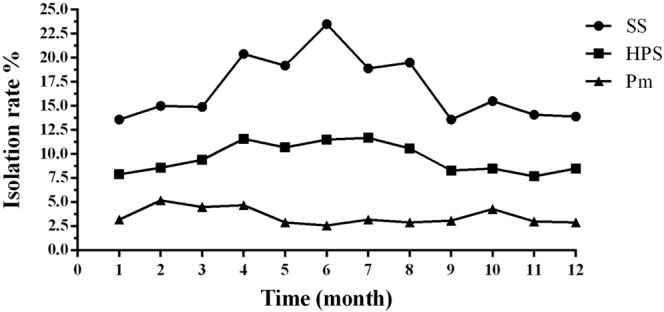
The seasonal prevalence characteristics of SS, HPS, and Pm. The accurate isolation rates of all bacterial pathogens in the different months from 1–12 were 13.6% (428/3151), 15.0% (388/2586), 14.9% (626/4207), 20.4% (836/4097), 19.2% (764/3992), 23.5% (899/3826), 18.9% (725/3843), 19.5% (697/3571), 13.6% (501/3673), 15.5% (517/3337), 14.1% (620/4389) and 13.9% (488/3503), respectively. As a whole, the differences in the isolation rates of SS, HPS, and Pm in 12 months were all significant (p < 0.05). Statistical analysis was performed using the χ2 test.

To investigate the seasonal prevalence characteristics of SS, HPS, and Pm, the number of samples and isolation rates of every month were calculated when samples were collected from 2013 to 2017. Results showed that the rates of isolation across the whole year of SS ranged from 13.6% to 23.5% with the highest isolation rate recorded in June (Fig. 4). While the monthly isolation rates of HPS ranged from 7.7% to 11.7% with the highest isolation rate recorded in July. By comparing the seasonal prevalence characteristics of SS and HPS, a similar phenomenon was found that they all had higher isolation rates from April to August. However, Pm showed higher isolation rates in February, March, April, and October ranging from 2.6% to 5.2% within a whole year (Fig. 5).

### The serotype of SS and HPS

**Figure 6:**
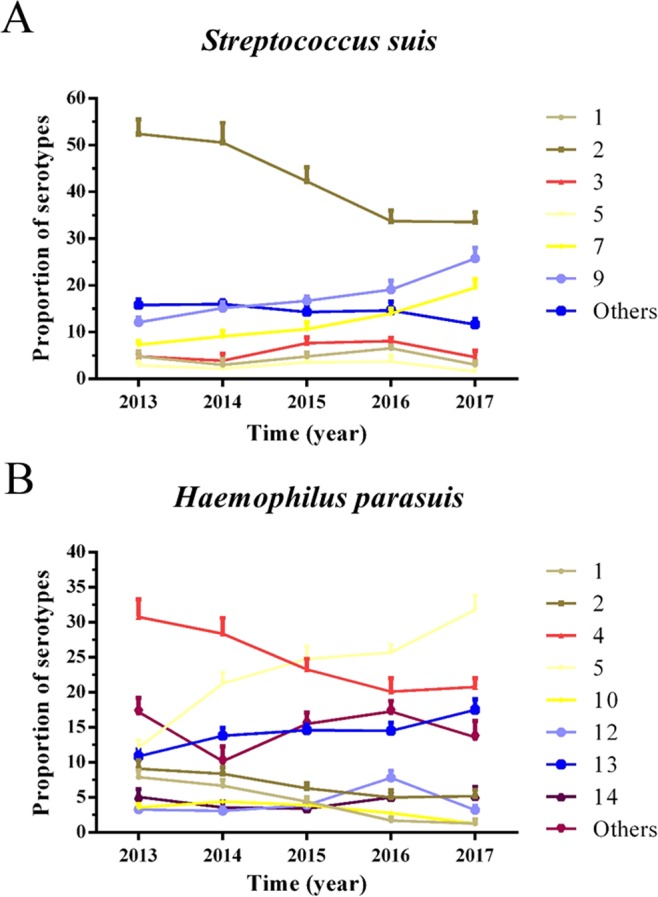
The proportion of different serotypes of SS (**A**) and HPS (**B**). The number of SS strains chosen for serotyping included 273, 231, 168, 136, and 128 strains from 2013 to 2017, respectively. Meanwhile, the number of HPS strains included 331, 225, 206, 179, and 154 strains from 2013 to 2017, respectively. As a whole, the differences in serotype 2 and 7 of SS from 2013 to 2017 were significant (p < 0.05), and the differences in serotype 4, 5 and 13 of HPS from 2013 to 2017 were significant (p < 0.05). Statistical analysis was performed using the χ2 test.

To better understand the most common bacterial pathogens in Chinese pig farms, a part of isolated SS and HPS were chosen to do serotyping experiments every year. The results showed that the major SS existed in Chinese pig farms were serotype 2, 9, 7, 3, 1, 5 from 2013 to 2017 and the highest isolated rate of SS was still serotype 2, but it declined sharply from 2013 (52.4%) to 2017 (33.6%). However, there were several other serotypes of SS increased over time. Such as serotype 7 (from 7.3% to 19.5%) and serotype 9 (from 12.1% to 25.8%) (Fig. 6A). Meanwhile, the major HPS existed in Chinese pig farms were serotype 4, 5, 13, 2, 1, 14, 10 and 12 from 2013 to 2017. Nevertheless, the proportion of different serotype of HPS changed obviously and the biggest change of HPS was serotype 4 (from 30.8% to 20.8%) and 5 (from 12.1% to 31.8%) which were the most popular serotypes of HPS in 2013 and 2017, respectively. At the same time, the proportion of HPS serotype 1 (from 7.9% to 1.3%), 2 (from 9.1% to 5.0%) and 10 (from 4.4% to 1.3%) declined, serotype 13 (from 10.9% to 17.5%) increased and serotype 12 and 14 did not show any change trends (Fig. 6B).

### Antibiotic resistance rates of SS, HPS, and Pm

**Figure 7:**
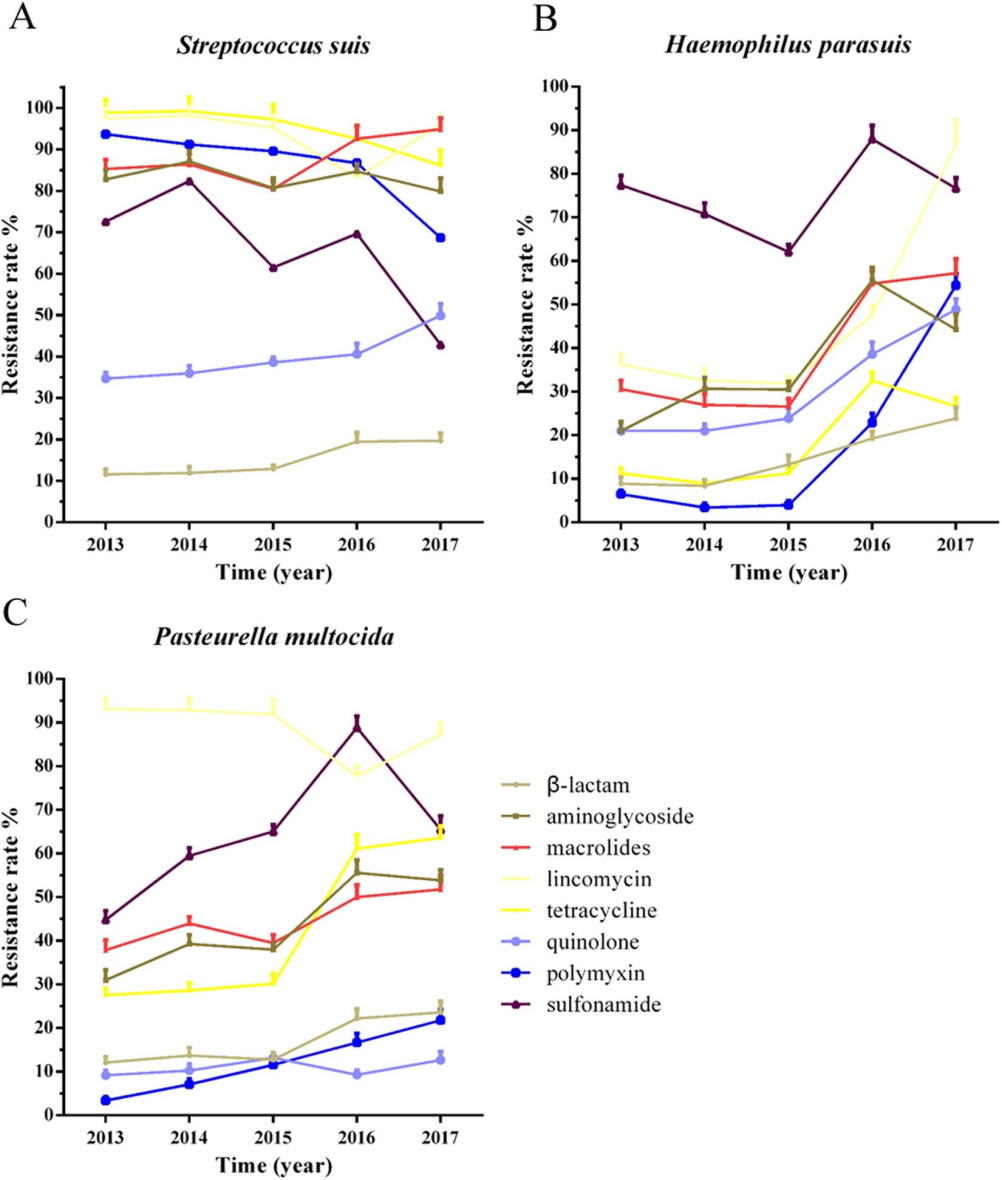
Antibiotic resistance rates of SS (**A**), HPS (**B**) and Pm (**C**) from 2013 to 2017. Antibiotics were chosen for antimicrobial susceptibility tests included β-lactam (cephradine, ceftriaxone, amoxicillin and ampicillin), aminoglycoside (streptomycin, gentamicin, spectinomycin, kanamycin, amikacin and neomycin), macrolides (spiramycin and azithromycin), lincomycin (lincomycin and clindamycin), tetracycline (doxycycline), quinolone (ofloxacin, ciprofloxacin and enrofloxacin), polymyxin (polymyxin B), and sulfonamide (trimethoprim) antibiotics. As a whole, the differences in resistance rates of SS, HPS, and Pm to all antibiotics from 2013 to 2017 were significant (p < 0.05), except resistance rates of SS to β-lactam and Pm to quinolone. Statistical analysis was performed using the χ2 test.

A total of 8 kinds of antibiotics have been chosen to do antimicrobial susceptibility tests including β-lactam (cephradine, ceftriaxone, amoxicillin and ampicillin), aminoglycoside (streptomycin, gentamicin, spectinomycin, kanamycin, amikacin and neomycin), macrolides (spiramycin and azithromycin), lincomycin (lincomycin and clindamycin), tetracycline (doxycycline), quinolone (ofloxacin, ciprofloxacin and enrofloxacin), polymyxin (polymyxin B) and sulfonamide (trimethoprim) antibiotics. Antibiotic resistance rates of SS, HPS, and Pm were presented in Table 3. Results showed that antibiotic resistance rates of SS to aminoglycoside, macrolides, lincomycin, tetracycline polymyxin, and sulfonamide antibiotics were all over 60% and it exhibited increasing antibiotic resistance rates in β-lactam (from 11.6% to 19.7%) and quinolone (from 34.7% to 49.9%) antibiotics (Fig. 7A). Meanwhile, the antibiotic resistance rates of HPS and Pm to different antibiotics are obviously lower than SS. But, HPS showed significant increasing antibiotic resistance rates to all kinds of antibiotics except sulfonamide which showed a strong antibiotic resistance rate to HPS (Fig. 7B). Just like HPS, Pm also had increasing antibiotic resistance rates to all kinds of antibiotics except lincomycin and quinolone antibiotics (Fig. 7C).

**Table 3:** Antibiotic resistance rates of SS, HPS, and Pm.

Bacteria/Antibiotic resistance rates	SS					HPS					Pm				
	2013 (95)	2014 (136)	2015 (182)	2016 (188)	2017 (217)	2013 (62)	2014 (89)	2015 (124)	2016 (83)	2017 (90)	2013 (29)	2014 (42)	2015 (43)	2016 (18)	2017 (55)
CE	0.063	0.059	0.055	0.112	0.129	0.000	0.011	0.065	0.048	0.044	0.000	0.000	0.047	0.056	0.036
CRO	0.189	0.162	0.143	0.207	0.217	0.129	0.045	0.113	0.193	0.167	0.310	0.310	0.256	0.389	0.400
AML	0.074	0.110	0.126	0.138	0.189	0.081	0.135	0.169	0.205	0.189	0.103	0.143	0.140	0.167	0.218
AMP	0.137	0.147	0.192	0.324	0.253	0.145	0.146	0.185	0.325	0.556	0.069	0.095	0.070	0.278	0.291
S	0.800	0.772	0.665	0.888	0.866	0.226	0.449	0.500	0.639	0.544	0.241	0.190	0.209	0.333	0.436
CN	0.832	0.941	0.907	0.830	0.788	0.161	0.213	0.274	0.446	0.333	0.207	0.571	0.558	0.611	0.509
SH	0.463	0.632	0.577	0.670	0.544	0.065	0.112	0.169	0.470	0.300	0.103	0.143	0.140	0.278	0.255
K	0.905	0.934	0.802	0.872	0.853	0.194	0.258	0.194	0.349	0.311	0.069	0.262	0.279	0.556	0.527
AK	0.979	0.978	0.951	0.840	0.903	0.306	0.427	0.363	0.542	0.422	0.552	0.524	0.512	0.778	0.636
N	0.989	0.971	0.940	0.979	0.839	0.306	0.382	0.331	0.892	0.744	0.690	0.667	0.581	0.778	0.873
SP	0.874	0.912	0.901	0.979	0.995	0.548	0.483	0.419	0.904	0.989	0.759	0.810	0.721	0.944	0.982
AZM	0.832	0.816	0.709	0.872	0.903	0.065	0.056	0.113	0.193	0.156	0.000	0.071	0.070	0.056	0.055
MY	0.989	0.993	0.962	0.851	0.935	0.274	0.213	0.210	0.470	0.744	0.897	0.905	0.884	0.778	0.836
DA	0.958	0.971	0.945	0.814	0.986	0.452	0.438	0.427	0.482	0.989	0.966	0.952	0.953	0.778	0.909
DO	0.989	0.993	0.973	0.926	0.862	0.113	0.090	0.113	0.325	0.267	0.276	0.286	0.302	0.611	0.636
OFX	0.326	0.360	0.385	0.335	0.341	0.081	0.124	0.210	0.217	0.556	0.034	0.071	0.116	0.056	0.109
CIP	0.421	0.441	0.429	0.473	0.770	0.403	0.348	0.274	0.651	0.533	0.172	0.143	0.186	0.111	0.109
ENR	0.295	0.279	0.346	0.410	0.387	0.145	0.157	0.234	0.289	0.378	0.069	0.095	0.093	0.111	0.164
PB	0.937	0.912	0.896	0.867	0.687	0.065	0.034	0.040	0.229	0.544	0.034	0.071	0.116	0.167	0.218
W	0.726	0.824	0.615	0.697	0.429	0.774	0.708	0.621	0.880	0.767	0.448	0.595	0.651	0.889	0.655

CE: Cephradine; CRO: Ceftriaxone; AML: Amoxycillin; AMP: Ampicillin; S: Streptomycin; CN: Gentamicin; SH: Spectinomycin; K: Kanamycin; AK: Amikacin; N: Neomycin; SP: Spiramycin; AZM: Azithromycin; MY: Lincomycin; DA: Clindamycin; DO: Doxycycline; OFX: Ofloxacin; CIP: Ciprofloxacin; ENR: Enrofloxacin; PB: Polymyxin B; W: Trimethoprim.

## Discussion

Based on samples collection information of geographical distribution, tissue sources and bacterial isolation rates, results had been concluded that SS, HPS, *E. coli*, Pm, APP, Bb, SE, and E. rhusiopathiae were ubiquitous in almost all Chinese pig farms (Figs 1 and 2). From the previous reports, we know that SS, HPS, Pm, APP, and Bb are the major bacteria which seriously influence the respiratory system of pigs^26^, *E. coli* and SE are the major pathogens of digestive system^4,5^, and E. rhusiopathiae will lead to endocarditis, cutaneous necrosis and arthritis^7^. They are all important pathogens that influence pig growth and productivity, and will lead to huge economic losses to the pig industry^27,28^. Though significant progresses have been made in the last few decades in reducing the prevalence of animal diseases, there is still an increasing concern over the economic losses associated with diseases that cause a reduction in production efficiency. Therefore, it is very important to know the prevalence characteristics and geographical distribution of these bacteria to control bacterial diseases efficiently.

This study fully evaluates the occurrence of common bacterial pathogens in Chinese pig farms. The results showed that the proportion of SS was highest among all isolated bacteria, followed by HPS, *E. coli*, Pm, APP, Bb, SE, and E. rhusiopathiae. Meanwhile, the order of the isolation rates of these bacterial pathogens was the same as the proportion of isolated bacteria (Fig. 3). Compared with the previous reports, higher isolation rates of SS, HPS, *E. coli*, and Pm were observed from pigs with respiratory diseases in agreement with some previous studies^29–31^. Whereas, lower isolation rates of Bb, APP, and E. rhusiopathiae were obtained^20,29,32^ (Fig. 4). On the other hand, although Pm and Bb did not show any obvious epidemic characteristics from 2013 to 2017, the changing trend of their isolation rates was almost the same which may associate with the characteristic that they always exist side by side and they are all the common pathogens of swine atrophic rhinitis^33^.

By analyzing the isolation rates of SS, HPS, and Pm among different months, the characteristics of their seasonal distribution were shown clearly. The isolation rates of SS and HPS were obviously higher in the warm season (from April to August) than the cold season because hot and wet climate is beneficial to the growth and transmission of SS and HPS^34^. However, the seasonal distribution of Pm was different with SS and HPS, Pm showed a significantly higher isolation rate from February to April (Fig. 5) which is almost the same with Bb as the report^20^. The reasons that lead to the phenomenon may be due to the discovery that Pm is always commonly existed with Bb which also has a high isolation rate from January to April^20,33^. On the other hand, because of cold and wet weather, the ventilation installations are always closed in pig farms to keep warm which contributes to the spread of Pm. Therefore, the climate is an important factor to monitor and control bacterial diseases.

It is reported that SS can be divided into at least 33 kinds of serotypes^35^ and HPS can also be divided into 15 kinds of serotypes^36^. However, their pathogenicity is different between different serotypes and they lack of cross-protection^37,38^. So, it is very important to know the clinical serotypes of SS and HPS for controlling bacterial diseases. Our serotyping results showed that the major serotypes SS existed in China were 2, 9, 7, 3, 1, 5 (Fig. 6A). But, it was different with other reports, especially, the decline of serotypes 3, 4, 8 and 1/2 and the increase of serotypes 7 and 9, which suggested the changing trend of different serotypes of SS in China^39^. On the other hand, SS of serotype 2 declined sharply from 2013 (52.4%) to 2017 (33.6%) which may be associated with the use of SS vaccine of serotype 2 in Chinese pig farms (Fig. 6A). A similar changing trend also appeared between different serotype of HPS. Based on serotyping results, serovars 4, 5 and 13 were still the major serotype of HPS which were the same as the reports before in China, North America, and Spanish^40–42^. Nevertheless, the percentage of different serotype of HPS varied largely and the main serotype of HPS changed from serotype 4 in 2013 (30.8%) to serotype 5 in 2017 (31.8%) which were all the main serotype of HPS existed in Chinese pig farms. There was also an obvious change in other serotypes (Fig. 6B). The main reasons that lead to the difference in serotype of SS and HPS may be due to the difference of environment, antibiotic, and the use of bacterial vaccines. Therefore, it is very important to change the serotype of the inactivated vaccines in controlling these diseases.

Because of the good effect of antibiotics in promoting growth and preventing infection, large amounts of antimicrobial agents are still being used in modern swine production around the world. This, in turn, would facilitate the emergence and development of antimicrobial resistance. So, it is very important to monitor antibiotic resistance rates of clinical pathogens^43^. In the experiments, we analyzed antibiotic resistance rates of SS, HPS, and Pm from 2013 to 2017. The results indicated that SS, HPS, and Pm all showed similar and very high antibiotic resistance rates to 8 kinds of detected antibiotics with the reports^15,44,45^. Thereinto, the antibiotic resistance rates of SS to these 8 kinds of antibiotics were all over 60% except β-lactam and quinolone. Meanwhile, these pathogens all displayed a rapid increase in antibiotic resistance rates of the common used antibiotics in China from 2013 to 2017, which maybe indicate irregular and excessive use of antibiotics. Based on the previous reports, the ways that some bacteria produce antibiotic resistance to aminoglycoside, macrolides, lincomycin, tetracycline, polymyxin, and sulfonamide mainly due to obtainment of exogenous resistance genes. However, the main reasons that lead to antibiotic resistance to β-lactam and quinolone are because of the mutation of drug targets, which is obviously more difficult than the first one^46^. Therefore, the difference of resistance mechanism maybe an important reason that the resistance rates of aminoglycoside, macrolides, lincomycin, and sulfonamide is obviously higher than that to β-lactam and quinolone in SS, HPS, and Pm. In total, reasonable application of antibiotics has become an urgent issue for the control of bacterial diseases.

## Conclusions

In summary, the identification and analysis results of 44175 collected samples showed that the main bacteria in Chinese pig farms were still SS, HPS, *E. coli*, and Pm. However, the isolation rates of different strains and serotypes of SS and HPS have obviously changed from 2013 to 2017. For example, SS, *E. coli*, APP, and SE displayed an increasing isolation rates. Nevertheless, HPS and E. rhusiopathiae displayed reversed results. The main serotypes of HPS had changed from serotype 4 to serotype 5, and the proportion of serotype 2 SS also decreased sharply from 2013 to 2017. Meanwhile, SS and HPS had an higher isolation rate in hot season. But, February, March, April, and October were the main seasons for the isolation of Pm. In addition, Antimicrobial susceptibility test indicated that SS, HPS, and Pm presented very high and increasing resistance rates to 8 kinds of common antibiotics. In conclusion, the study provides us very detailed information on the prevalence and antimicrobial susceptibilities of several main bacteria in China from 2013 to 2017, which help us to understand, prevent and control bacterial diseases of Chinese pig farms.

## Supplementary information

Supplementary Table 1
