# Supplementary material for: Prevalence and antimicrobial susceptibilities of bacterial pathogens in Chinese pig farms from 2013 to 2017
Source: Sci Rep. 2019 Jul 9;9:9908. doi: 10.1038/s41598-019-45482-8 (PMC6616368; doi:10.1038/s41598-019-45482-8)
Supplement: Supplementary file 1 — Supplementary Table 1 [file 41598_2019_45482_MOESM1_ESM.docx]

**Prevalence and antimicrobial susceptibilities of bacterial pathogens in Chinese pig farms from 2013 to 2017**

Bingzhou Zhang^a^, Xugang Ku^a^, Xuexiang Yu^a^, Qi Sun^a^, Hao Wu^a^, Fangzhou Chen^a^, Xiaoqian Zhang^a^, Long Guo^b^, Xibiao Tang^b^, Qigai He^a^*

^a^Division of Animal Infectious Diseases, State Key Laboratory of Agricultural Microbiology, College of Animal Sciences and Veterinary Medicine, Huazhong Agricultural University, Wuhan 430070, P.R. China.

^b^The Diagnostic Center for Animal Disease of Huazhong Agricultural University. Wuhan 430070, P.R. China.

* Corresponding author. Tel.: +86-27-8728-6974; Fax: +86-27-8728-7288; E-mail addresses: [he628@mail.hzau.edu.cn](mailto:he628@mail.hzau.edu.cn) (Q.G. He)

Table 1. Primers used in this study.

| Strains | Gene | Name | Sequence (5’ → 3’) | Amplicons size (bp) | References |
| --- | --- | --- | --- | --- | --- |
| *Streptococcus suis*  serotype primers | | | | | 30 |
| SS1 | *cps1I* | *cps1IF*  *cps1IR* | TCTTATAACAGGCGTCAAAACA  ATCGGTATAAAAGCAAGACACA | 153 |  |
| SS2 | *cps2I* | *cps2IF*  *cps2IR* | TTCGTATTAACTTACTTGGCGT  TAAATCCCCATATGCCAAATCC | 363 |  |
| SS3 | *cps3L* | *cps3LF*  *cps3LR* | ACATCCATTGCAGGAGTAGT  TGCAGTTCCAAAATTCTTCGT | 210 |  |
| SS4 | *cps4K* | *cps4KF*  *cps4KR* | TGATATTGGCTATCTTTTGGGG  TTCCCCCTTCAAATAAACTCTG | 542 |  |
| SS5 | *cps5L* | *cps5LF*  *cps5LR* | AGGTATGTCTTCTTATTCGCAG  ATAATCCCTCCTGATACTAGGC | 428 |  |
| SS6 | *cps6I* | *cps6IF*  *cps6IR* | TGGTGTCTTTCTACCTGCAA  TCACCAAGATACGTGAACCA | 705 |  |
| SS7 | *cps7L* | *cps7LF*  *cps7LR* | AAAATTCGTTCCATTGTAGGTG  TGAAGTTGAAGCTGGTGATAAA | 609 |  |
| SS8 | *cps8K* | *cps8KF*  *cps8KR* | ATCGCTTCAAATAAGGTAGGAG  TGTAGGCCGTAATATCAACAAA | 268 |  |
| SS9 | *cps9J* | *cps9JF*  *cps9JR* | TGAAAGTAGGTATATCTCAGCA  AAAGAATTGAATCCCACCTGAG | 809 |  |
| SS10 | *cps10M* | *cps10MF*  *cps10MR* | CTATCACTACCACGGAATGC  TAACCGTCCGTCTAGAATGT | 303 |  |
| SS11 | *cps11N* | *cps11NF*  *cps11NR* | ATTGTTACGATTTGGGCGAT  GAACCCCATGTAGTTATGGC | 512 |  |
| SS12 | *cps12J* | *cps12JF*  *cps12JR* | CATGGGAACTGTACAGGATAAG  CCACCTTACTACCTGTTTTACC | 171 |  |
| SS13 | *cps13L* | *cps13LF*  *cps13LR* | GCTTGTAGCGAATTTTGGTATT  CCATTAGATGTATTTGCTCCCA | 741 |  |
| SS15 | *cps15K* | *cps15KF*  *cps15KR* | ACCTACTCAAGAACATCCTTTC  GTAACTAAAACAGCAAACGTCA | 458 |  |
| SS16 | *cps16I* | *cps16IF*  *cps16IR* | ATCAACAAACATTTTCGAGGAC  GCTGAATAATAGATTCGTCCTGT | 223 |  |
| SS17 | *cps17O* | *cps17OF*  *cps17OR* | TTGCCGTATAAGGTCTTAGTTG  ATCTGACGGTAAATGTTCTCTG | 380 |  |
| SS18 | *cps18N* | *cps18NF*  *cps18NR* | ATAGGCTGTACTTTGATAACCG  AGCCTATCGCTCAAAAACTTAT | 310 |  |
| SS19 | *cps19L* | *cps19LF*  *cps19LR* | ATTATTATAGGGCAAAGCAGGG  ATCGTACACAACAAAACGATTC | 674 |  |
| SS20 | *cps20I* | *cps20IF*  *cps20IR* | TAATCGTTGCCTTTGAGCAT  CGCTATATAAGGAAACCTCGG | 938 |  |
| SS21 | *cps21P* | *cps21PF*  *cps21PR* | TGGCAGACTTCTTTTCTCAC  CCTGTAGCGCCTCATAAAAC | 858 |  |
| SS22 | *cps22K* | *cps22KF*  *cps22KR* | AGGATCGGTAAGTTTAGGTACA  ATGCAGTAAAACACGAAAACAA | 158 |  |
| SS23 | *cps23J* | *cps23JF*  *cps23JR* | TATTATAGTCCGATGCAAGCAG  ATGAGAACGAAACGGAATAGTT | 461 |  |
| SS24 | *cps24M* | *cps24MF*  *cps24MR* | GATAGCAATGTAATCCAATCGC  GTAGGTTCCCCTAGTAAGAAGT | 204 |  |
| SS25 | *cps25M* | *cps25MF*  *cps25MR* | ATTGAGTCCTTTTACTGGTAGC  TACTGAGCTACATAATCCCACA | 390 |  |
| SS26 | *cps26P* | *cps26PF*  *cps26PR* | CAAAATTCCTGGATTAACGCTT  CGATCTGAGGACTTATCAAGAA | 315 |  |
| SS27 | *cps27K* | *cps27KF*  *cps27KR* | GTGGTTTTGGAGGATATTTTCG  ATTGAGATAAACTACTCCGTGC | 530 |  |
| SS28 | *cps28L* | *cps28LF*  *cps28LR* | GGGCACTTGTTTTACTTCCT  GCCAAGTAATACCCTACCTG | 896 |  |
| SS29 | *cps29L* | *cps29LF*  *cps29LR* | AAAGTGCCTATTCTGGGATTTT  TAAAGGCAACTTCCACATTGTA | 263 |  |
| SS30 | *cps30I* | *cps30IF*  *cps30IR* | TTGGGCTTGTAAATAGTGAGAG  CGATTAGATAAGCGCATTTGTT | 625 |  |
| SS31 | *cps31L* | *cps31LF*  *cps31LR* | CATATGTTTTCGTGGGGAGT  GTGATGAAAACATCGTTGGTAG | 1006 |  |
| SS33 | *cps33K* | *cps33KF*  *cps33KR* | GAGTTGCGACCTATTATTCTCA  GAATTGAACAACGACTGCAATA | 731 |  |
| *Haemophilus parasuis*  serotype primers | | | | | 31 |
| HPS2 | *wzx* | *wzxF*  *wzxR* | CTAACAAGTTAGGTATGGAGGGTTTTGGTG  GGCACTGAATAAGGGATAATTGTACTG | 295 |  |
| HPS3 | *glyC* | *glyCF*  *glyCR* | CATGGTGTTTATCCTGACTTGGCTGT  TCCACATGAGGCCGCTTCTAATATACT | 650 |  |
| HPS4 | *wciP* | *wciPF*  *wciPR* | GGTTAAGAGGTAGAGCTAAGAATAGAGG  CTTTCCACAACAGCTCTAGAAACC | 320 |  |
| HPS5 or 12 | *wcwK* | *wcwKF*  *wcwKR* | CCACTGGATAGAGAGTGGCAGG  CCATACATCTGAATTCCTAAGC | 450 |  |
| HPS6 | *gltI* | *gltIF*  *gltIR* | GATTCTGATGATTTTTGGCTGACGGAACG  CCTATTCTGTCTATAAGCATAGACAGGAC | 360 |  |
| HPS7 | *funQ* | *funQF*  *funQR* | CTCCGATTTCATCTTTTCTATGTGG  CGATAAACCATAACAATTCCTGGCAC | 490 |  |
| HPS8 | *scdA* | *scdAF*  *scdAR* | GGAAGGGGATTACTACTACCTGAAAG  CTCCATAGAACCTGCTGCTTGAG | 650 |  |
| HPS9 | *funV* | *funVF*  *funVR* | AGCCACATCAATTTTAGCCTCATCA  CCTTAAATAGCCTATGTCTGTACC | 710 |  |
| HPS10 | *funX* | *funXF*  *funXR* | GGTGACATTTATGGGCGAGTAAGTC  GCACTGTCATCAATAACAATCTTAAGACG | 790 |  |
| HPS11 | *amtA* | *amtAF*  *amtAR* | CCATCTCTTTAACTAATGGGACTG  GGACGCCAAGGAGTATTATCAAATG | 890 |  |
| HPS13 | *gltP* | *gltPF*  *gltPR* | GCTGGAGGAGTTGAAAGAGTTGTTAC  CAATCAAATGAAACAACAGGAAGC | 840 |  |
| HPS14 | *funAB* | *funABF*  *funABR* | GCTGGTTATGACTATTTCTTTCGCG  GCTCCCAAGATTAAACCACAAGCAAG | 730 |  |
| HPS15 | *funI* | *funIF*  *funIR* | CAAGTTCGGATTGGGAGCATATATC  CCTATATCATTTGTTGGATGTACG | 550 |  |

30 Liu, Z. *et al.* Development of Multiplex PCR Assays for the Identification of the 33 Serotypes of Streptococcus suis. *Plos One* **8**, e72070 (2013).

31 Howell, K. J. *et al.* Development of a Multiplex PCR Assay for Rapid Molecular Serotyping of Haemophilus parasuis. *Journal of clinical microbiology* **53**, 3812-3821 (2015).
